# Mechanical properties of trabeculae and osteocyte morphology change significantly in different areas of the necrotic femoral head

**DOI:** 10.3389/fcell.2023.1250070

**Published:** 2023-09-26

**Authors:** Min-Cong He, Jia-Qing Tian, Xiao-Ming He, Peng Yang, Tian-Ye Lin, Qing-Wen Zhang, Wei He, Qiu-Shi Wei

**Affiliations:** ^1^ Guangdong Research Institute for Orthopedics and Traumatology of Chinese Medicine, The Third Affiliated Hospital, Guangzhou University of Chinese Medicine, Guangzhou, China; ^2^ The Third Clinical Medical College, Guangzhou University of Chinese Medicine, Guangzhou, China; ^3^ The Third Affiliated Hospital, Guangzhou University of Chinese Medicine, Guangzhou, China

**Keywords:** necrotic femoral head, osteocyte network, trabecular bone, mechanical properties, pathogenesis

## Abstract

**Background:** Osteonecrosis of the femoral head is a complex hip ailment. The precise changes in bone tissue during the disease’s onset remain unclear. It is vital to assess both the quantity and quality of the trabecular state in a necrotic femoral head.

**Aim:** This study aims to identify and compare the ultrastructural changes in osteocyte morphology and nanomechanical characteristics within various regions of necrotic femoral heads.

**Methods:** Between December 2016 and May 2023, we gathered ten necrotic femoral heads from patients and five femoral heads from cadavers. The samples from the necrotic femoral heads were categorized into three areas: necrotic, sclerotic, and normal. Our assessment methods encompassed hematoxylin and eosin staining, sclerostin (SOST) immunohistochemistry, micro-computed tomography, nanoindentation, and acid-etched scanning electron microscopy. These techniques enabled us to examine the SOST expression, trabecular microstructure, micromechanical properties of trabeculae, and modifications in osteocyte morphology at the ultrastructural level.

**Results:** The protein level of SOST was found to be lower in the sclerotic area. In the necrotic area, decreased values of bone volume fraction, trabecular thickness, and trabecular number and an increased value of trabecular separation were found. Conversely, in the sclerotic area, higher mean values of bone volume fraction, trabecular number, and trabecular thickness and lower trabecular separation indicated significant changes in the structural characteristics of trabeculae. Compared with the healthy area, the elastic modulus and hardness in the sclerotic area were significantly higher than those in the necrotic, normal, and control areas, while those in necrotic areas were significantly lower than those in the healthy area. The number of osteocytes tended to increase in the sclerotic area with more canalicular cells compared to the healthy area and control group.

**Conclusion:** These results imply that the stress distribution within the sclerotic area could potentially lead to enhanced trabecular quality and quantity. This effect is also reflected in the increased count of osteocytes and their canaliculars. It is plausible that the sclerotic trabecular bone plays a pivotal role in the repair of necrotic femoral heads.

## Introduction

Osteonecrosis of the femoral head (ONFH) is a refractory hip condition that predominantly impacts individuals aged 30 to 50. In the United States alone, around 10,000 to 20,000 new cases are identified annually, with approximately 5%–12% of total hip arthroplasties conducted annually to address ONFH (Guggenbuhl et al., 2021; Li et al., 2021). Femoral head collapsing and hip osteoarthritis can lead to serious hip pain and limited range of motion (Gasbarra et al., 2015). The precise mechanisms underlying the changes in bone tissue within the femoral head during pathogenesis remain elusive (Wang et al., 2022). Both quantity and quality play pivotal roles in assessing the state of trabecular structure within a necrotic femoral head. Micro-CT can delineate bone quantity, while the mechanical properties significantly impact the quality of bone tissue (Judex et al., 2003). Nanoindentation is a relatively ideal technique to investigate the mechanical properties at the extremely small tissue level (such as single trabeculae). Nanoindentation offers a means to evaluate the quasi-static and dynamic mechanical properties of trabeculae, enabling the computation of bone tissue’s pre-yield characteristics. Furthermore, nanoindentation facilitates the comparison of the elastic modulus or hardness of neighboring bone tissue within close proximity (Busa et al., 2005).

Osteocytes, together with the lacunocanalicular system, can regulate bone mass by sensing mechanotransduction and providing responses to mechanical loading (Cabahug-Zuckerman et al., 2016; Javaheri et al., 2014; Jing et al., 2014). By expressing various cytokines, osteocytes can also mediate osteoclasts’ activity (Nakashima et al., 2011). Several studies have reported that canalicular numbers are closely related to bone quality through the technique of acid-etching scanning electron microscopy (SEM) (Milovanovic et al., 2013). Factors such as aging can significantly decrease the density of the osteocyte network, and a higher density of osteocyte networks indicates higher bone material quality (Kerschnitzki et al., 2013). Furthermore, poor bone conditions such as microcrack accumulation can be found in low-lacunar-density human bone samples (Vashishth et al., 2000). All the studies suggest the osteocyte network plays an important role in the maintenance of healthy bone tissue.

By integrating immunohistochemistry, micro-CT, nanoindentation, and acid-etching SEM techniques, this study aims to comprehensively analyze both the quantity and quality of trabecular bone tissue. It also seeks to explore the osteocyte network’s status within various regions of necrotic femoral heads, providing deeper insights into the significance of sclerotic areas and the underlying pathological alterations associated with ONFH.

## Materials and methods

### Sample collection

Necrotic femoral heads were obtained from individuals who underwent total hip arthroplasty for late-stage ONFH, and a control group consisted of five femoral heads from cadavers. The recruitment of patients took place at the Third Affiliated Hospital of Guangzhou University of Chinese Medicine between December 2016 and May 2023. The inclusion criteria for ONFH patients encompassed the following: a) the absence of a hip trauma history, b) late-stage ONFH necessitating total hip arthroplasty, and c) willingness to provide informed consent.

### Femoral head section

After collection during total hip arthroplasty, the samples were coronally sectioned from posterior to anterior using the EXAKT-Cutting and Grinding System (Norderstedt, Germany), resulting in 8 mm-thick sections. For analysis, the central four sections, which best represented the femoral head, were selected. All samples were preserved in 10% formalin. In the necrotic head group, at least two bone cubes from each of three distinct areas, necrotic, sclerotic, and healthy, were cut within a single section. Control group bone samples were acquired from the central area of the femoral head in each section. Micro-CT scans were performed on all bone cubes, with at least one cube from each area undergoing HE staining and immunohistochemistry analysis for SOST. The remaining bone samples were embedded in epoxy resin, awaiting subsequent nanoindentation and acid-etched SEM procedures.

### HE staining and immunohistochemistry of SOST

After the micro-CT scanning was completed, the bone samples were immersed in 10% formalin at room temperature for more than 24 h. Subsequent to decalcification in 10% EDTA and dehydration, the samples underwent embedding in paraffin wax. Embedded specimens were cut into 5 μm sections and stained with HE. The specimens were washed with tris-buffered saline and Tween 20, and then, the endogenous peroxidase activity was quenched by 3% hydrogen peroxide for 30 min. Antigen retrieval was performed in citrate buffer for 10 min on an 85°C hot plate. TBST-5% bovine serum albumin (BSA) was used to block non-specific reactivity at room temperature for 30 min. Rabbit anti-human SOST (ab85799, Abcam, Cambridge, MA, United States) antibodies (dilution 1:1250) were used to incubate together with representative slides overnight at 4°C. A secondary biotinylated antibody (ab96899, DyLight^®^ 488 goat anti-rabbit IgG) was applied for 30 min at room temperature. Slides were counterstained with hematoxylin at room temperature for 6 min. The images from HE staining and immunohistochemistry were captured by using a microscope (BX53, Olympus).

### Micro-CT scanning

Micro-CT with a spatial resolution of 9 nm was used to analyze the trabecular bone at the quantitative level (Skyscan 1076, Skyscan, Belgium). Bone volume fraction (BV/TV), trabecular thickness (Tb.Th), trabecular number (Tb.N), and trabecular separation (Tb.Sp) calculated by CTAn (CTAn, Skyscan, Belgium) were selected to evaluate the trabecular bone microarchitecture parameters.

### Nanoindentation test

The bone cubes underwent a sequential ethanol dehydration process, starting with a concentration of 70% ethanol, followed by 80%, 90%, and finally, 100%. Each concentration was maintained for a period of 2 days. Notably, no preliminary decalcification step was carried out before this dehydration process. After the ethanol dehydration, the bone samples were embedded in epoxy resin and left to cure for a minimum of 24 h. The samples were polished with silicon papers (Buehler–CarbiMet, Illinois, United States) of number 320 until the surfaces of interest were exposed. Subsequently, the surfaces were further polished with grit numbers of 600, 1200, 2400, and 4000 in order to acquire a smooth surface. Finally, polycrystalline diamond suspensions (Buehler MetaDi™ Supreme) of roughness 3 μm, 1 μm, 0.25 μm, and 0.05 μm were used to polish the samples before nanoindentation. The mechanical properties such as elastic modulus and hardness were calculated on a microscale in the axial direction by nanoindentation (Hysitron Triboindenter TI-950, Minneapolis, Minnesota). All the indentation procedures were finished with a Berkovich tip. In order to maintain consistency in each sample, multiple trabeculars (at least three in each bone cube) were chosen for indents. The tip area function was evaluated from indentation analysis on fused quartz, and drift rates in the system were measured before each indentation by standard indentation testing procedures. When the indentation began, a preload of 2 μN was applied. The indentation procedure, which was 50 s in total, was combined by a 10 s loading period at a constant loading rate of 100 μN/s, a 30 s constant load segment at the peak of 1,000 μN, and then finally, a 10 s retracting in the unloading segment at a constant unloading rate of 100 μN/s. A 20%–90% portion of the unloading curve was extracted to calculate the elastic response.

### Acid-etching SEM

Following the completion of the nanoindentation test, the surfaces of the epoxy-embedded samples underwent an acid-etching procedure. This involved exposing the samples to 37% phosphoric acid for a duration of 10 s. Subsequently, the samples were thoroughly rinsed with distilled water, undergoing five washes of 2 min each. After the sodium hypochlorite wash at a concentration of 5% for a duration of 10 min, the samples were once again subjected to a series of distilled water washes, with each wash lasting 2 min. This distilled water washing process was repeated five times. Following these washes, the samples were left to air-dry overnight. Subsequently, the surface of the samples was coated with a layer of gold palladium; this process is used to prepare the samples for SEM imaging. Magnifications of 150X, 500X, 1K X, 2K X, 5K X, and 10K X were used to visualize bone microarchitectures and individual osteocytes. The osteocyte canalicular number at 10K X magnification and the oteocyte number at 150X magnification were manually marked and quantified using Image-Pro Plus 6.0 (Media Cybernetics, United States).

### Ethical approval

This study adheres to the ethical standards set forth by the Review Board on Human Research of the Faculty of Medicine and aligns with the principles outlined in the Declaration of Helsinki. Additionally, it received the necessary approval from the ethics committee of the Third Affiliated Hospital of Guangzhou University of Chinese Medicine (No. PJ-KY-20220420-013), ensuring compliance with ethical guidelines and oversight.

### Statistical analysis

A one-way ANOVA test was performed for comparing the parameters of all three necrotic, sclerotic, and healthy areas together in the control group. IBM statistical software SPSS 22.0 was used for the one-way ANOVA statistical analysis. The significance level was set at 0.05.

## Results

We harvested 10 necrotic femoral heads as the ONFH group and five femoral heads from cadavers as the control group from patients. The age of the ONFH group is in the range of 42–60 (49.4 ± 5.5), and that of the control group is in the range of 42–73 (53.2 ± 12.5) (*p* > 0.05). Patients in the ONFH group were all in the late stage of ONFH (ARCO stage 3 or 4).

### HE staining and immunohistochemistry of SOST

**FIGURE 1:**
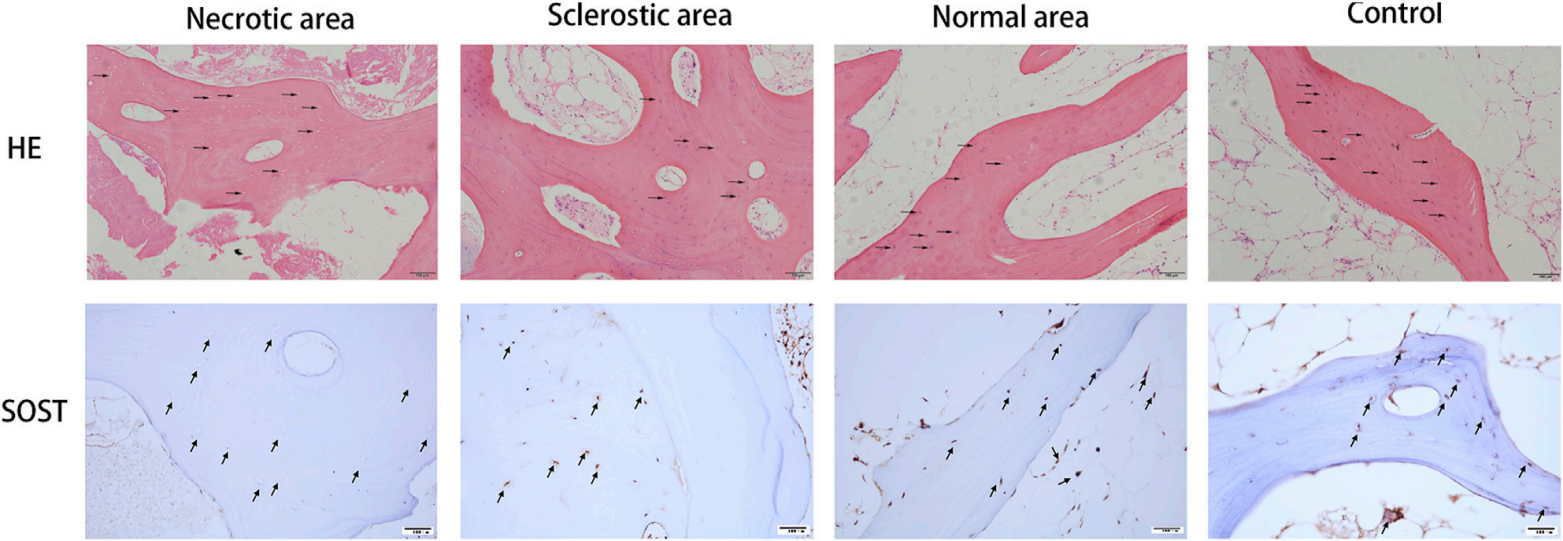
Results from HE staining and immunohistochemistry of SOST. Osteocytes were missing in the lacunae (black arrow). SOST was barely detectable in the necrotic area. SOST-positive cells in the sclerotic area were significantly decreased compared to the healthy area and control group (black arrow).

The results from HE staining and immunohistochemistry of SOST are shown in Figure 1. Samples from the normal area and control group display a healthy and complete trabecular bone structure, and the lacunae are filled with osteocytes. Conversely, results from the necrotic area display a lack of osteocytes within the lacunae. SOST presence is observed within the trabecular bone, but its detection is notably limited in the necrotic area due to the absence of osteocytes. Additionally, the area positive for SOST in the sclerotic region exhibits a significant reduction when compared to both the healthy area and the control group.

### Micro-CT evaluation

**FIGURE 2:**
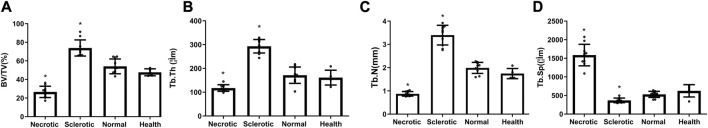
**(A–D)** Decreased values of BV/TV, Tb.Th, and Tb.N and an increased value of Tb.Sp were found in the necrotic area, while higher mean values of BT/TV, Tb.N, and Tb.Th and lower Tb.Sp were detected in the sclerotic area (*significantly different from the control group).

The quantity of bone trabecular in necrotic, sclerotic, and healthy areas shows a significant difference (Figure 2). Trabecular bone in the necrotic area reveals poor quantity with decreased BV/TV, Tb.Th, and Tb.N and increased Tb.Sp. Trabecular bone in the sclerotic area undergoes significant changes with higher BT/TV, Tb.N, and Tb.Th and lower Tb.Sp.

### Nanoindentation test

**FIGURE 3:**
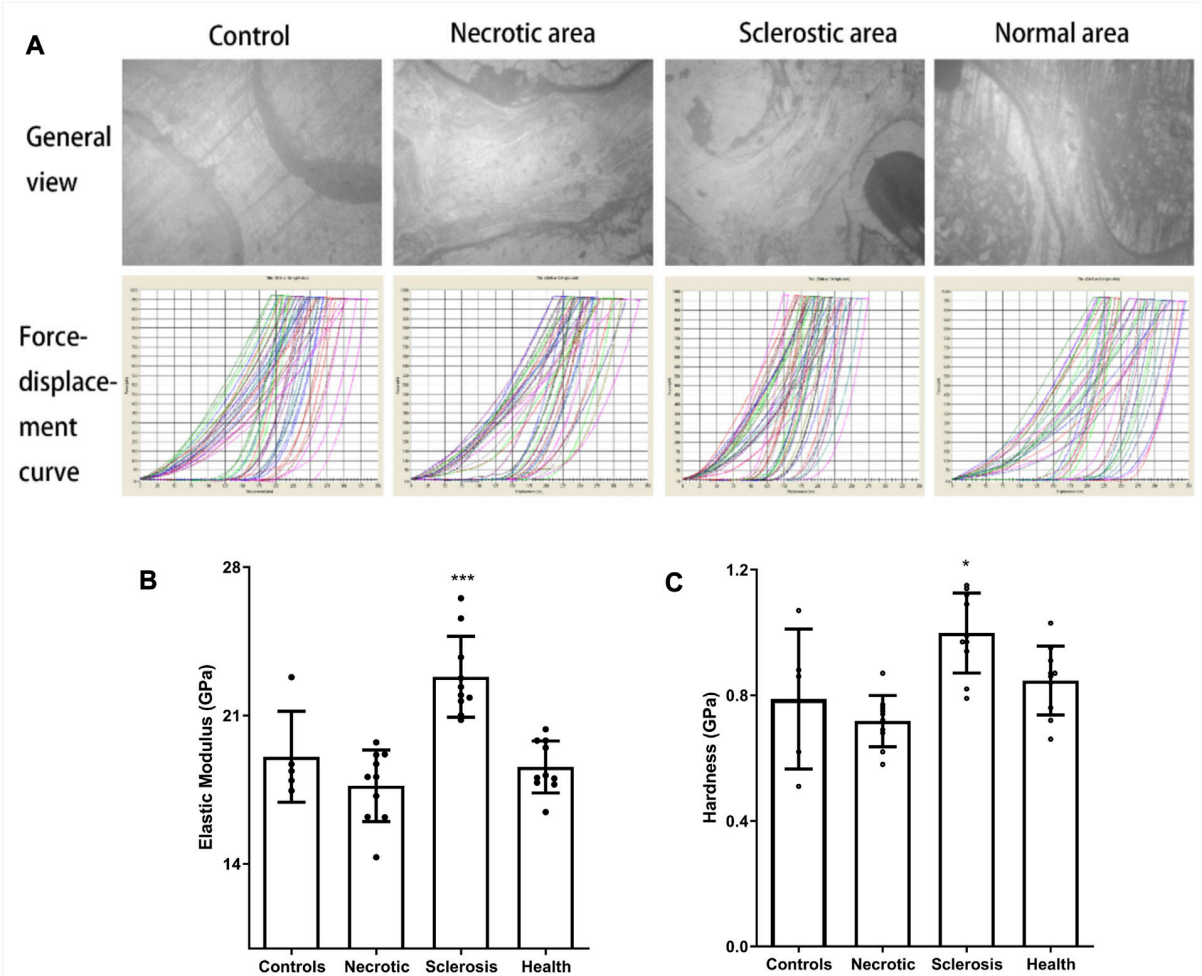
**(A)** Images from the microscope in nanoindentation and force-displacement curves. **(B, C)** Elastic modulus and hardness in the sclerotic area were significantly higher than those in the necrotic, normal, and control areas. Trabecular elastic modulus in the necrotic area was significantly lower than that in the healthy area (*p < 0.05; ***p < 0.01).

The images from the microscope in nanoindentation and force-displacement curves are shown in Figure 3A. In contrast to the healthy area, the elastic modulus and hardness within an individual trabecular structure from the sclerotic region exhibit notable increases, which are statistically significant when compared to the results observed in the necrotic, normal, and control areas. The trabecular elastic modulus within the necrotic area is lower than that observed in the healthy area, but the difference is not significant (Figures 3B, C).

### Acid-etched SEM

**FIGURE 4:**
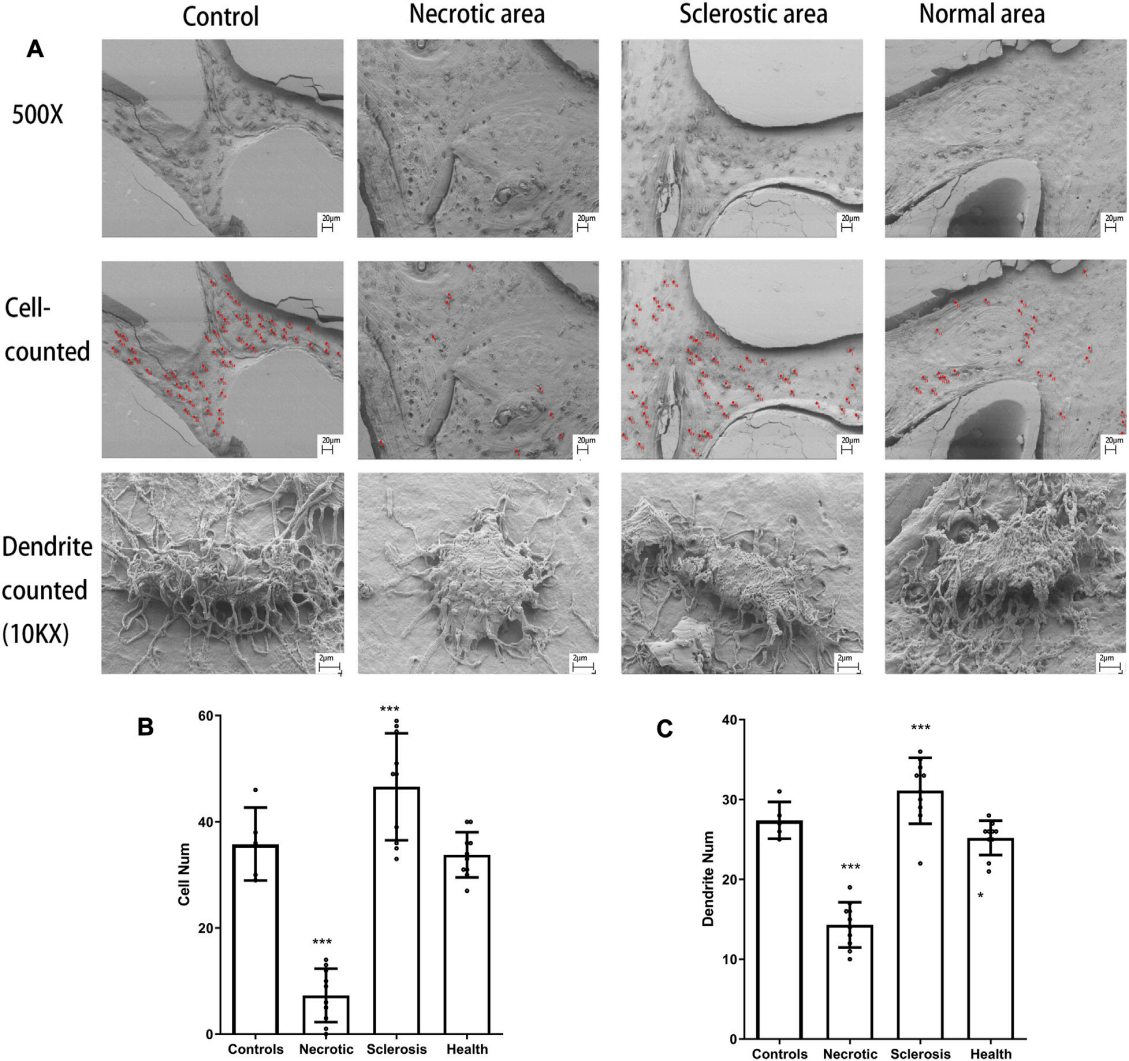
**(A)** Osteocytes are marked with red spots. **(B, C)** Both the number of osteocytes and the number of canaliculars in the sclerotic area were significantly increased. A lower number of osteocytes and canaliculars in the necrotic area was found (***p < 0.01).

The unmarked and marked images in different tissues are shown in Figure 4A. Both the number of osteocytes and the number of canaliculars in the sclerotic area are significantly increased (Figures 4B, C). A lower number of osteocytes are found in the necrotic area. Moreover, a low number of canaliculars around osteocytes are observed in the remaining osteocytes in the necrotic area (Figures 4B, C).

## Discussion

Our findings indicate substantial alterations in bone microstructure, nanomechanical properties, and the ultrastructural morphology of osteocytes in ONFH when compared to the control group. The microarchitecture of trabeculae in the necrotic area and expression of SOST had remarkable changes. Trabeculae showed stronger mechanical properties, and there was a significant increase in the cell number of osteocytes and canaliculars in the sclerotic area.

Bone consists of a composition comprising a collagenous matrix and rigid crystals. The presence of collagen contributes to the toughness of bone, reducing its brittleness and guarding against fractures. Simultaneously, minerals within bone provide stiffness, ensuring resistance to deformation under loading conditions. Following Wolff’s Law, bone tissues alter their size and shape to accommodate mechanical stresses and adapt accordingly (Teichtahl et al., 2015). Weight bearing that generates ground reaction and muscle contraction forces together become hip joint loading (Sievanen, 2010; Wang et al., 2022). According to a study using a strain gauge and telemetric data transmission in total hip arthroplasty, the real-time hip contact forces during walking can increase nearly 2.5 times body weight during the stance (weight-bearing) phase of walking but less than the body weight during the swing (non-weight-bearing) phase (Heller et al., 2001). Trabeculae are densely distributed in areas experiencing elevated mechanical stress, serving as an effective mechanism to enhance bone strength within the femoral head. Femoral head collapse, which was an important signal of deterioration of ONFH, was caused by the changes in trabecular bone inside the necrotic femoral head (Pascart et al., 2022). Multiple studies from animal models or human samples reported that the microstructure of trabeculae from necrotic tissue was significantly different from that from normal bone tissue (Ma et al., 2017). The deterioration of trabeculae numbers may relate to the apoptosis or necrosis of osteocytes in the necrotic area. As most osteocytes died, bone resorption was markedly enhanced, and the necrotic bone was replaced by bone remodeling (Kennedy et al., 2014). Furthermore, proinflammatory cytokines, including tumor necrosis factor-α (TNF-α) and interleukin (IL)-6, which induce Rankl expression in osteoblasts and lead to osteoclastogenesis, are released from lacunae to the bone surface through canaliculi (Komori, 2016). In addition, the released ATP through Panx1 channels recruits macrophages and monocytes and enhances the membrane fusion of osteoclast precursor cells to form multinucleated osteoclasts and strengthen osteoclast survival (Cheung et al., 2016). It can explain the results where the quantity of trabeculae in the necrotic area is significantly lower than that in other areas. The expression of SOST in different areas was evaluated by immunohistochemistry. SOST was chosen to, at least in part, reveal the mechanical condition of different areas. SOST expression in osteocytes is downregulated by loading and upregulated by unloading (Pietrzyk et al., 2017). In a previous *in vivo* study, SOST-deficient models were resistant to unloading bone loss and did not indicate decreased bone formation in unloading conditions (Lin et al., 2009). The reduction in SOST expression in osteocytes is required for enhanced bone formation by mechanical loading (Tu et al., 2012). Moreover, SOST-positive cells were decreased by loading in the compressive stimuli when compared to the tensional stumili (Moriishi et al., 2012). SOST expression is likely suppressed in a compressively loaded condition because stress distributions are concentrated along the sclerotic boundary (Karasuyama et al., 2015). Chen et al. (2018) reported a lower serum SOST in patients with late-stage ONFH and indicated that reduced expression of SOST may play a key role in the collapse process of ONFH and be predictive of the disease progression of ONFH. Furthermore, Wang et al. (2014) observed an elevation in osteoblast activity within the sclerotic area, along with an increase in osteoclast activity within the necrotic area. These findings suggest a potential association with alterations in macroscopic mechanical strength.

Trabeculae within the sclerotic area exhibited distinct differences, encompassing both quantity and quality aspects. Hengsberger et al. (2005) reported that the overall macro-mechanical properties of bone are not solely determined by micromechanical properties. The augmented quantity and improved quality can be attributed to stress redistribution, a phenomenon that takes place from the outset and during the advancement of ONFH (Brown et al., 1983). According to multiple finite element analyses (Brown et al., 1983), stress concentration was found at the interface of necrotic and sclerotic areas or trabecular bone below. To adapt to the increased stress, bone remodeling began in the sclerotic area in multiple aspects. In the aspect of bone quantity, trabeculae not only became thicker and denser but also appeared disordered (increased values of BV/TV and Tb.Th and decreased Tb.Sp). In the aspect of bone quality, the biomechanical properties were strengthened (higher elastic modulus and hardness). The main reason for these improvements in biomechanical properties is not completely clear yet. A previous study by Zaino et al. (2010) indicated that it might be related to the formation of mixed tissues (including chondroid tissue or woven bone tissue). Combined with our results of SOST expression, micro-CT, and SEM, the bone-healing process in the sclerotic area may precipitate increases in the elasticity modulus and hardness of the trabeculae. The mechanical properties, such as elasticity modulus and hardness, of trabecular bone in the necrotic area were not significantly different compared with the healthy area or healthy control group. Hence, we propose that the diminished mechanical properties alone may not be the sole factor contributing to femoral head collapse. The degradation of macro-mechanical changes could also arise from alterations in microstructure. Similar findings have been observed in osteoporosis animal models. Even though the bone mass or microstructure improved after treatment with fibroblast growth factor or parathyroid hormone injection, the elasticity modulus or hardness of trabecular in the proximal tibia was not changed (Guo et al., 2000; Lane et al., 2003).

Based on the acid-etched SEM findings, we further corroborated the significant increase in both osteocyte numbers and canalicular counts within the sclerotic area. To the best of our knowledge, our study stands as the pioneer in finding the ultrastructural changes of osteocytes within a necrotic femoral head. Synchrotron radiation-based X-ray computed tomography (SRCT) is another advanced technique to observe the osteocytes and canalicular processes in 3D conditions. Compared to the resolution of SEM (nearly 1 nm), SRCT only provides images with a resolution of 700 nm (Schneider et al., 2011). Furthermore, the acid-etching method is an effective technique to confirm osteocyte networks and canalicular structures. Osteocytes, together with their canalicular networks connecting to other osteocytes, have the ability to sense mechanical stimulation and mediate bone anabolic/catabolic responses to mechanical stimulation (Klein-Nulend et al., 2012). In the present study, bone tissues from the necrotic, sclerotic, and healthy areas and healthy control bone were utilized for SEM analysis. More osteocytes with increased numbers of canaliculars arising directly from the cell membrane were found in the sclerotic area, whereas fewer osteocytes with decreased numbers of canaliculars were found in the necrotic area. Canaliculars are formed in the process of the transition of osteoblasts to osteocytes. Compression stimuli can activate osteoblasts to produce a bone matrix (van Tol et al., 2020). Thus, it is possible that the osteoblasts under compressive conditions can transition into osteocytes with more bone matrix than those in the less-loading condition. The increased canalicular numbers indicate that the connection between the osteoblasts and osteocytes during the transition is greater in the sclerotic area than in the other areas. In the molecular biological aspect, fluid shear stress, direct cellular deformation, or other mechanical stimulation generated by stress distribution can be translated to load-induced molecular signals through the upregulated expression of bone morphogenetic proteins (BMPs), Wnts, prostaglandin E2 (PGE2), and NO (Santos et al., 2009). Connexin (Cx) 43 hemichannels, which can be opened by mechanical loading, were reported to regulate osteogenesis molecules (Li et al., 2013). It has been demonstrated that the osteocyte canalicular can not only transduct the mechanical stimulation and open the Cx 43 hemichannels on the osteocyte’s body but also transport loading-related signals to neighboring osteocytes (Bellido, 2014).

## Limitation

Our study has several limitations. First, patients with ONFH who request total hip replacement treatment are always in an advanced stage of the disease. Specimens at earlier stages were not collected due to ethical issues, and the necrotic femoral head samples tested in our study were in stages III or IV. Therefore, in spite of the results of SOST expression and SEM, including previous finite element analysis, which together indirectly reveal that mechanical stress concentrations may exist in the sclerotic area and may be related to bone repair, we do not have the evidence to provide a thorough explanation of the mechanical aspects of bone reconstruction in the sclerotic region. Furthermore, the cells or factors regulating mechanical properties in the sclerotic region are not clear yet. More molecular biological studies are needed to clarify the particular processes. At last, changes in osteocytes, trabecular structure, and biomechanical properties of the necrotic femoral head indicated abnormalities in cell biology, which we need to explore in further studies.

## Conclusion

The findings indicate that stress distribution within the sclerotic area might lead to an enhanced abundance of high-quality trabeculae, along with elevated osteocyte numbers and canaliculars. This underlines the potential significance of sclerotic trabecular bone in the repair of necrotic femoral heads.

## Data Availability

The raw data supporting the conclusion of this article will be made available by the authors, without undue reservation.

## Abbreviations

SOST, sclerostin; SEM, scanning electron microscopy; ONFH, osteonecrosis of the femoral head; THA, total hip arthroplasty; HE, hematoxylin and eosin; BV/TV, bone volume fraction; Tb.Th, trabecular thickness; Tb.N, trabecular number; and Tb.Sp, trabecular separation.
